# Laboratory confirmed puerperal sepsis in a national referral hospital in Tanzania: etiological agents and their susceptibility to commonly prescribed antibiotics

**DOI:** 10.1186/s12879-019-4324-5

**Published:** 2019-08-05

**Authors:** Raymond Kiponza, Belinda Balandya, Mtebe V. Majigo, Mecky Matee

**Affiliations:** 1 Department of Obstetrics and Gynaecology, Muhimbili University of Health and Allied Sciences, Dar es Salaam, Tanzania; 2 Department of Microbiology and Immunology, Muhimbili University of Health and Allied Sciences, Dares Salaam, Tanzania

**Keywords:** Puerperal sepsis, Antimicrobial susceptibility, Blood culture, Endocervical swabs, Aetiology, Tanzania

## Abstract

**Background:**

In most developing countries, puerperal sepsis is treated empirically with broad spectrum antibiotics due to lack of resources for culture and antibiotics susceptibility testing. However, empirical treatment does not guarantee treatment success and may promote antimicrobial resistance. We set to determine etiological agents and susceptibility pattern to commonly prescribed antimicrobial agents, among women suspected of puerperal sepsis, and admitted at Muhimbili National Hospital.

**Methods:**

Hospital based cross-sectional study conducted at tertiary hospital from December 2017 to April 2018. The study recruited post-delivery women suspected with puerperal sepsis. Socio- demographic, clinical and obstetric information were collected using structured questionnaire. Blood and endocervical swab samples were collected for aerobic culture. Blood culture bottles were incubated in BACTEC FX40 (Becton–Dickinson, Sparks, MD, USA). Positive blood cultures and cervical swabs were inoculated onto sheep blood agar, MacConkey agar, chocolate agar and Sabouraud’s dextrose agar, incubated aerobically at 37 °C for 18–24 h. Antimicrobial susceptibility was determined by Kirby-Bauer disc diffusion method.

**Results:**

A total of 197women were recruited, of whom 50.3% had spontaneous vaginal delivery, while 49.2% had caesarean section. Bacteraemia was detected in 22 (11.2%) women, along with 86 (43.6%) isolated from endocervical swabs. Gram-negative bacilli were the predominant isolates detected in 92(46.7%) cases. Majority of the isolates were *E. coli* 68(61.8%) followed by *Klebsiella* spp. 22(20.0%). *E. coli* were highly susceptible to meropenem (97.0%), while resistance to ceftriaxone, ampicillin and ceftazidime was 64.7, 67.6 and 63.2%, respectively. *Klebsiella* spp. were susceptible to meropenem (86.4%) and resistant to ceftriaxone (77.3%), gentamicin (86.4%), ampicillin (81.8%) and ceftazidime (86.4%). *Staphylococcus aureus* isolates were 100% susceptible to clindamycin. The proportion of extended spectrum beta lactamase producers among gram-negative bacilli was 64(69.6%) and 53.8% of *S. aureus* isolates were resistant to methicillin.

**Conclusion:**

In this study puerperal sepsis was mostly caused by *E. coli* and *Klebsiella* spp. Causative agents exhibited very high levels of resistance to most antibiotics used in empiric treatment calling for review of treatment guidelines and strict infection control procedures.

## Background

Puerperal sepsis accounts for 15% of maternal deaths worldwide [1]. In Africa, puerperal sepsis is the second leading cause of maternal morbidity and mortality, accounting for more than 10% of maternal deaths [1]. On the other hand, the rate of puerperal sepsis has declined significantly in high-income countries. For example, in the United States puerperal sepsis occur in only 5.5% of vaginal deliveries and 7.4% of caesarean section deliveries [2, 3].

The vast majority of puerperal sepsis are due to infection of the genital tract by pathogens that colonize the cervix and vagina, gain access to amniotic fluid and invade the devitalised uterine tissues [4]. Maternal anaemia, prolonged labour, excessive number of vaginal examinations during labour as well as prolonged rupture of membranes, increases the risk of puerperal sepsis. However, caesarean delivery is the single most important risk factor whereas peri–procedural antibiotic prophylaxis reduces the risk significantly [5]. Variety of bacterial pathogens have been implicated in causing puerperal sepsis including: wide range of anaerobes like *Peptostretococcus*, C*lostridia*, *Pseudomonas* and *Bactericides fragilis* and facultative aerobes such as *Escherichia coli*, *enterococci, Klebsiella* spp., beta-haemolytic *Streptococci*, and *staphylococci* [4, 6–10].

In most developing countries, puerperal sepsis is treated empirically with broad spectrum antibiotics due to lack of resources for culture and antibiotics susceptibility testing [10]. This practice does not ensure treatment success and possesses an increased risk for development of antimicrobial resistance to commonly prescribed antibiotics [11]. The antimicrobial susceptibility patterns of causative agents of puerperal sepsis differ widely across geographic areas and with time [6, 7, 10, 12]. Unfortunately, most of patients in settings with limited resources have limited treatment options, and the consequence of antibiotic resistance to the affordable antimicrobial agents is very significant.

We carried out this study at Muhimbili National Hospital (MNH), the National Referral Hospital in Tanzania. The purpose was to identify the common bacterial pathogens causing puerperal sepsis and to document susceptibility pattern of antibiotics commonly prescribed at the facility. The finding from this study might help to improve empirical treatment at health facilities in which cultures and antimicrobial susceptibility testing are not performed routinely.

## Methods

### Study design, setting and population

This was hospital based cross-sectional study conducted from December 2017 to April 2018 at MNH, the largest tertiary hospital in Tanzania. MNH receives referred patients from regional and district hospitals in Dar es Salaam and nearby regions for specialized obstetrics services. The institutional protocol for management of puerperal sepsis requires laboratory investigations for blood culture and either endocervical swabs or urine depending on presenting features. Empiric treatments using broad spectrum antibiotics are promptly administered after specimen collection. The protocol allows provision of prophylactic antibiotics to pregnant women undergoing caesarean section.

The study employed convenient sampling to recruit women admitted in maternity wards for postnatal care with clinical diagnosis of puerperal sepsis based on World Health Organization (WHO) criteria [1]. Eligible were women with genital tract infection between the day of rupture of membranes and the 42nd day postpartum. In addition, coinciding with fever of 38.5 °C or higher, along with one or more of the following features: pelvic pain, abnormal vaginal discharge, abnormal odour or discharge, or a delay in the reduction of uterine size. We excluded patients who have been on antibiotics for seven days or more from time of encounter for recruitment eligibility. Only patients who gave written informed consent were consecutively enrolled. The estimated minimum sample size of 128 was calculated using Kish Lisle formula, considering 9.2% maternal mortality due to puerperal sepsis at MNH [13], 95% confidence interval and 5% margin of error.

### Data collection

A structured data collection tool was used to gather socio- demographic, clinical and obstetric information as well as physical examination and laboratory finding. Trained research assistant conducted interviews; physical examinations were performed and findings recorded by qualified obstetrician. Empiric treatment was initiated soon after specimen collection for women without prior treatment. However more than half of the women, most of them referred from other facilities had already started treatment before specimen collection.

### Specimen collection and transportation

Two sets of 10 ml of blood were collected from each patient by a trained phlebotomist while observing aseptic technique. Each blood sample was inoculated in a separate blood culture bottle, BACTEC Aerobic vials (Becton–Dickinson, Sparks, MD, USA) using a sterile need and was immediately transported to Bacteriology Research Laboratory at Muhimbili University of Health and Allied Sciences (MUHAS) within 2 h of collection.

Endocervical swab samples were carefully collected from the endocervix gator by a qualified obstetrician during pelvic speculum examination avoiding vaginal contamination. The specimens were placed into Ames transport medium (Medical Wire Tran swab Ames, UK) and transported to the Microbiology laboratory at MUHAS for processing within 6 h of collection.

### Isolation and identification of bacteria

Blood culture bottles were incubated aerobically at 37 °C in BACTEC FX40 (Becton–Dickinson, Sparks, MD, USA) blood culture system according to the instructions of the manufacturer. Positive blood cultures were sub-cultured onto Sheep Blood Agar (SBA) MacConkey Agar (MCA) and Chocolate Agar (CA) and incubated aerobically at 35- 37 °C for 24 h. Similarly, endocervical swabs were inoculated on CA, SBA, MCA and Sabouraud’s Dextrose Agar (SDA) and incubated aerobically at 35- 37 °C for 24 h.

Preliminary identification of bacteria isolates was done based on cultural characteristics such as colonial morphology, haemolysis pattern on SBA, changes in physical appearance in differential media, Gram staining and biochemical tests. Identification of gram-negative organisms was carried out by performing a series of biochemical tests including Kligler Iron Agar, Sulphur indole motility, Simon’s citrate agar and urease test. Gram-positive bacteria were identified by conventional methods.

### Antimicrobial susceptibility testing

Antimicrobial susceptibility testing (AST) was performed using the Kirby-Bauer disk diffusion method according to Clinical Laboratory Standard Institute (CLSI) [14]. Briefly, homogenous colonial suspension was prepared from pure culture comparable to 0.5 McFarland turbidity standards and inoculated on Mueller-Hinton agar. The plates were incubated at 37 °C for 18–24 h and zone of inhibition were interpreted according to CLSI guidelines. The following antibiotic disks (Oxoid, UK) were tested on both gram negative and Gram-positive bacteria; ciprofloxacin (5 μg), gentamicin (10 μg) and ampicillin (10 μg). Clindamycin (2μg) and Penicillin G (10 IU) were used for only gram-positive bacteria isolates while ceftriaxone (30 μg), ceftazidime (30 μg) and Meropenem (10 μg) on gram-negative organisms.

Methicillin resistant *Staphylococcus aureus* (MRSA) was determined by disc diffusion test using cefoxitin (30 μg) disc on Mueller Hinton Agar, incubated and maintained at 33–35 °C for 24 h. Zone of inhibition ≤21 mm was considered a positive result for MRSA strain. Extended spectrum beta-lactamase (ESBL) production was screened by disc diffusion method on Mueller-Hinton Agar using ceftriaxone (30 μg). All isolates with zones of inhibition of < 22 mm for ceftazidime and ≤ 25 mm for ceftriaxone were confirmed by Modified Double Disc Synergy Test. [15] Amoxicillin-clavulanate (30 μg) was placed in the centre of the plate, ceftazidime (30 μg) and cefotaxime (30 μg) discs were placed 20 mm apart. Any distortion or increase in the zone towards the disc of amoxicillin-clavulanate was considered as positive for ESBL production.

## Results

A total of 214 women admitted at MNH between December 2017 and April 2018 suspected of having puerperal sepsis, were assessed for recruitment eligibility. After assessment 197(92.1%) met eligibility criteria and consented to be recruited in the study. Seventeen women did not meet the WHO criteria for diagnoses of puerperal sepsis. On admission participants had following features: - 100% had fever of 38.5 °C or higher; 194(98.5%) pelvic pain, 194 (98.5%) abnormal vaginal discharge, 196 (99.5%) abnormal odour or discharge and 196 (99.5%) had delay in reduction of uterine size (< 2 cm/day during the first 8 days after delivery).

As shown in Table 1, majority of women were; aged 20–35 years (75.6%), had primary education (67.5%), married (87.8%) and had no employment (54.8%). Most of them (77.1%) had parity of 1 to 3; moreover about 33% were primiparas. Most of the women 105 (53.3%) were referred from peripheral hospitals, including 5(2.5%) who delivered at home. Almost equal proportion of women had spontaneous vaginal delivery (50.3%) and caesarean section (49.2%), only one had an assisted vaginal delivery. Most of the patients (53.3%) were seen between 8 and 14 days post-delivery. About 20% of the babies had unfavourable outcome, either a low Apgar score (4.5%), still birth (7%) or early neonatal death (8.5%).

**Table 1:** Social demographic characteristics of 197 women with features of puerperal sepsis

Characteristics	N (%)
Age(years)	
< 20	15 (7.6)
20–35	149 (75.6)
> 35	33 (16.8)
Education level	
No formal education	30 (15.2)
Primary	133 (67.5)
Secondary	33 (16.8)
College/university	1 (0.5)
Marital status	
Single	24 (12.2)
Married	173 (87.8)
Occupation	
Unemployed	108 (54.8)
Petty trader	84 (42.6)
Salaried employment	5 (2.5)
Parity	
1	65 (33.0)
2	52 (26.4)
3	35 (17.8)
4	25 (12.7)
> 4	20 (10.2)
Mode of delivery	
Spontaneous vaginal delivery	99 (50.3)
Caesarean section	97 (49.2)
Assisted vaginal delivery	1 (0.5)
Place of delivery	
MNH	92 (46.7)
Referred from peripheral hospitals	105 (53.3)
Time post-delivery (days)	
< 8	24 (12.2)
8–14	105 (53.3)
15–21	50 (25.4)
22–28	14 (7.1)
> 28	4 (2.0)
Neonatal outcome	
Normal	160 (80)
Low apgar score	9 (4.5)
Still birth	14 (7.1)
Early neonatal death	17 (8.5)

Among 197 patients with puerperal sepsis, 143 (72.6%) had prolonged labour, whilst 12 (6.1%) required obstetric manoeuvre for delivery. A total of 22 (11.2%) received interventional surgical procedures, among them 17/22 (77.3%) had subtotal hysterectomy, while 5/22 (22.7%) had total hysterectomy. Substantial number of participants 32 (16.2%) were admitted at intensive care unit. The maternal death happened to 15 (7.6%).

As shown in Table 2, the most commonly prescribed combination of antibiotics on admission or prior to admission was ceftriaxone and metronidazole (52.3%), followed by meropenem and metronidazole (29.9%), and lastly ampicillin and metronidazole (2.5%). Antibiotics regimen was mainly influenced by drugs availability and physician discretion. However, 109 (55.3%), mostly women referred from other facilities, had already started antibiotic before admission. (Table 2).

**Table 2:** Combination of antibiotics used in empiric treatment of the participants

Antibiotics used	N (%)
Ceftriaxone + metronidazole	103 (52.3)
Ceftriaxone + metronidazole + gentamicin	30 (15.2)
Ampicillin + metronidazole	5 (2.5)
Meropenem+ metronidazole	59 (29.9)

A total of 22 (11.2%) blood cultures were positive, with *Klebsiella* spp. (31.8%) the most predominant bacteria, followed by *E .coli* (27.3%) *and S. aureus* (22.7%), while *Pseudomonas* and *Enterococci* each accounted for 9.1%. About 68% (15/22) of pathogens were isolated from patients who had prior treatment. For endocervical culture, majority of isolates were *E. coli* (72.1%) followed by *Klebsiella* spp. (17.4%), and *S. aureus* (9.3%), among them 50% were isolated from patients with prior treatment. (Table 3).

**Table 3:** Bacterial isolated from blood and endocervix of women with puerperal sepsis

Pathogen	Blood cultures		Endocervical swabs		Total Isolates
	Total Isolates N (%) ^a^	From patients with Prior treatment N (%) ^b^	Total Isolates N (%) ^a^	From patients with Prior treatment N (%) ^b^	
*Klebsiella* spp	7 (31.8)	4 (57.1)	15 (17.4)	9 (60.0)	22
*E. coli*	6 (27.3)	4 (66.7)	62 (72.1)	29 (46.8)	68
*Pseudomonas* spp	2 (9.1)	2 (100.0)	–	–	2
*Enterococci*	2 (9.1)	2 (100.0)	–	–	2
*S. aureus*	5 (22.7)	3 (60.0)	8 (9.3)	4 (50.0)	13
Yeast cells	–	–	1 (1.2)	1 (100.0)	1
Total	22 (100.0)	15 (68.2)	86 (100.0)	43 (50.0)	108

^a^ Percentage within total isolates, ^b^ percentage within specific isolate

As summarized in Table 4, *E. coli* isolates were highly susceptible to meropenem (95.6%), moderately sensitive to gentamicin (61.8%) and ciprofloxacin (55.9%), while resistance to ceftriaxone and ceftazidime was 64.7 and 63.2%, respectively. *Klebsiella* spp. were susceptible to meropenem 86.4% and moderately susceptible to ciprofloxacin (50%), while resistant to ceftriaxone (77.3%), gentamicin (86.4%), and ceftazidime (86.4%). *S. aureus* was susceptible to clindamycin (100%). *Pseudomonas* was totally resistant to ceftriaxone, meropenem, and gentamicin, only moderately susceptible to ciprofloxacin, while *enterococci* were totally resistant to ciprofloxacin, ampicillin and penicillin. Majority of resistant pathogens were isolated from patients who had antibiotic treatment before enrolment. (Table 4).

**Table 4:** Resistance pattern of the isolates to the commonly prescribed antibiotics

Bacteria	N	Percent of resistant isolates							
		CEF	MER	CIP	CLI	GEN	AMP	PEN	CTZ
*Klebsiella*	22	77.3	13.6	50		86.4	81.8	–	86.4
*E. coli*	68	64.7	2.9	44.1	–	38.2	67.6		63.2
*Pseudomonas*	2	100	100	50		100	100	–	100
*Enterococcus*	2	–	–	100	–	–	100	100	–
*S. aureus*	13	–	–	38.5	0	15.4	–	69.2	–

Key: *CEF* Ceftriaxone, *MER* Meropenem, *CIP* Ciprofloxacin, *CLI* Clindamycin, *GEN* Gentamicin, *AMP* Ampicillin, *PEN* Penicillin, *CTZ* Ceftazidime

As shown in Table 5, proportion of ESBL producing Gram-negative bacteria was 69.6%, while 53.8% of *S. aureus* were resistant to methicillin, referred to as MRSA. More than half (51.6%) of ESBL producing pathogens were isolated from patients who received antibiotics prior to specimen collection. (Table 5).

**Table 5:** Proportion of MRSA & ESBL producing Bacteria

Proportion	# of test	Total Positive N (%)	From patients with prior treatment N (%)
ESBL	92	64 (69.6)	33 (51.6)
MRSA	13	7 (53.8)	3 (42.7)
TOTAL	105	71	36

## Discussion

We investigated, among women hospitalized at a National Referral Hospital in Dar es Salaam suspected of puerperal sepsis, putative etiological agents and their susceptibility to commonly prescribed antibiotics. Most of the women were referred from peripheral hospitals and half had spontaneous vaginal delivery, but an almost equal percentage had a caesarean section. There was a significant delay in seeking care as most of the women were seen between 8 and 14 days post-delivery. Sadly, about one in every five had unfavourable obstetric outcome, either low Apgar score, stillbirth or early neonatal death which is significantly higher compared to 5 in 100 of general unfavourable obstetrics outcome in the same facility.

In this study bacteraemia was detected in 11.2% of the women and the most common isolates were *Klebsiella* spp. (31.8%), followed by *E. coli (*27.3%) and *S. aureus* (22.7%), with *enterococci* and *Pseudomonas*, each accounting for 9.1% of the total isolates. For endocervical swabs, majority of isolates were *E. coli* (72.1%) followed by *Klebsiella* spp. (17.4%), and *S. aureus* (9.3%). In general, there was a predominance of Gram-negative bacilli, accounting for 89.4% of all isolates; whereas Gram-positive cocci were isolated in only 12.5%.of the cases.

Our findings are similar to those of other studies, showing predominance of Gram negative bacteria among cases of puerperal sepsis [10], and predominance of *Klebsiella* and *E. coli*, followed by *Staphylococcus* [16]. However, our findings differ with findings of a study in Nigeria, where the leading isolate was *S. aureus*, followed by *E. coli*, and *K. pneumoniae* [7] and study in Boston United State, where *E. coli* was predominant in blood culture followed by *Bacteroides* species [17]. Collectively, these studies show variations in aetiology of puerperal sepsis [8, 9, 11, 17–19], which could be due to differences in the type of patients such as age, immune status or underlying conditions as well as variations in bacteriological techniques used in sample collection and cultivation of the bacteria [16].

In some studies, like the present one, only facultative techniques were used, while in others both anaerobic and facultative techniques were used [6, 17]. The variation in isolation of *S. aureus* could be partly due to differences in the levels of asepsis in sample collection and processing, leading to possible contamination [10]. Our study did not isolate group A and B streptococci, which are reported in recent studies to contributed to puerperal sepsis [17, 20]. The lack of group A and B streptococci could be contributed by the fact that our study recruited slightly more than half already started antibiotics for one to six days with regimens containing ampicillin.

In this study, *E. coli and Klebsiella* spp.*,* which were the predominant etiological agents, were highly susceptible to meropenem (86.4 and 95.6%) and moderately susceptible to gentamicin and ciprofloxacin, and resistant to ceftriaxone, ceftazidime and gentamicin. *S. aureus* was highly sensitive to clindamycin. These differences could be attributed in part, to the prescription tendencies at the hospital. The findings indicate that ceftriaxone is the most commonly prescribed drug, accounting for 67.5% of all prescriptions, while meropenem was prescribed to 29.9% of the patients. Although ampicillin and gentamicin were prescribed less frequently in these patients, these antibiotics are very widely used for treatment of other infections and resistance to them is very high [10]. Worth mentioning, majority of resistant pathogens were obtained from patients referred from lower facility, who started treatment before enrolment. This gives an indication that most of antibiotic used for empirical treatment are not effective.

Majority of Gram-negative bacteria were ESBL producers and most of the *S. aureus* isolates were resistant to methicillin. Similar observations were seen in a study conducted at the same hospital by Manyahi et al. [12], indicating high level of multidrug resistance pathogens. We also noted that *Pseudomonas* was totally resistant to ceftriaxone, meropenem, gentamicin, and moderately sensitive to ciprofloxacin. *Enterococci* were totally resistant to ciprofloxacin, ampicillin and penicillin. We note that all *Pseudomonas* and *Enterococci* were isolated from patients who had started treatment few days before admission. This high level of antimicrobial resistant underlines significant challenges in successful treatment of puerperal sepsis using the current treatment guidelines at MNH.

Based on observed level of resistance, we suggest a change from ceftriaxone to meropenem for empiric treatment of Gram-negative agents of puerperal sepsis at MNH. Similarly we advocate the use of clindamycin for *S. aureus* and *Enterococci*. The high resistance of the isolates to ampicillin means it should be excluded in empiric treatment of puerperal sepsis, while the variable effectiveness of ciprofloxacin, ceftriaxone, ceftazidime and gentamicin imply a restricted use unless laboratory results are available to support their use.

This study has a number of limitations. We were not able to isolate strict anaerobes due to limitations placed on the collection and processing of specimens for anaerobic bacteriology. For the same reason we could not test the usefulness of metronidazole, which is currently used for empiric treatment of women suspected of puerperal sepsis. Secondly, some of the isolates were found in small numbers that limit the power of making treatment recommendations. Furthermore, convenience sampling method used in this study raises the possibility of biases inherent to such a sampling technique. Finally, majority of participants were admitted for postnatal care already stated treatment, which might have influenced our finding on aerologic distribution and antimicrobial resistance pattern.

The major strength of this study was that it was conducted in the largest tertiary care hospital in Tanzania, drawing patients from various parts of the country.

## Conclusion

*E. coli, Klebsiella and S. aureus* are the most common causative agents of puerperal sepsis at MNH. The pathogens exhibit high levels of resistance to common prescribed antibiotics that prompt urgent review of the management of puerperal sepsis at the facility. We are also advocating for improvement of infection control measures to reduce the incidence of puerperal sepsis among patients admitted in this hospital. Further studies are recommended to examine anaerobic cause and neonatal infection associated with puerperal sepsis.

## Data Availability

All relevant data generated and analysed during this study are available from the corresponding author on reasonable request.

## Abbreviations

AST: Antimicrobial susceptibility testing
CA: Chocolate Agar
CLSI: Clinical Laboratory Standard Institute
ESBL: Extended spectrum beta-lactamase
MCA: MacConkey Agar
MNH: Muhimbili National Hospital
MRSA: Methicillin Resistant *Staphylococcus aureus*
MUHAS: Muhimbili University of Health and Allied Sciences
SBA: Sheep Blood Agar
SDA: Sabouraud’s Dextrose Agar
WHO: World Health Organization
